# Noninvasive Cell Population Profiling of Normal and Dysplastic Cervical Biofluids by Multicolor Flow Cytometry as a Promising Tool for Companion Diagnostics

**DOI:** 10.3390/cancers17203328

**Published:** 2025-10-15

**Authors:** Christoph Berger, Wolf Dietrich, Manuela Richter, Florian Kellner, Christian Kühne, Katharina Strasser

**Affiliations:** 1 Valdospan GmbH, Technopark 1D, 3430 Tulln an der Donau, Austria; 2 RDP Pharma AG, Amriswilerstrasse 51, 8590 Romanshorn, Switzerland; 3 Department of Gynecology and Obstetrics Tulln, Karl Landsteiner University of Health Sciences, Alter Ziegelweg 10, 3430 Tulln an der Donau, Austria

**Keywords:** biofluids, flow cytometry, pap smear, cervical intraepithelial lesion, cervical cancer, companion diagnostics

## Abstract

**Background/Objectives**: Cervical Pap smears are routinely used to detect cellular abnormalities as a cervical cancer screening tool and to assess the presence of HPV for risk stratification of the disease. Here, we aimed to extend the applications of this sampling procedure by combining it with multicolor flow cytometry to characterize cell populations across cervical cancer disease stages. **Methods**: Cervical Pap smears from 30 patients with various disease stages ranging from normal to intraepithelial neoplasia up to treated cancers were analyzed as biofluids using multicolor flow cytometry. Individual samples were evaluated, and statistical analyses were performed over all sample stages. Cancer cell lines (CaSki, SiHa, HeLa, A549, U2OS) were examined as tumor cell controls. **Results**: Cervical biofluids were subdivided into cell populations according to their scattering properties and the expression of specific biomarkers: EpCAM and cytokeratin 8 for epithelial cells from tumors as well as healthy ectocervical and endocervical regions, and CD45 for immune cells. Discrimination of tumor cells was facilitated with cancer cell lines. Statistical analysis revealed that the composition of cell populations differs among disease stages, whereas treated cancer samples were consistently associated with a reduction in squamous epithelial cells and an increase in immune cells compared to normal samples. **Conclusions**: Herein, we identified the major cell populations in cervical biofluid samples and demonstrated that this method can detect changes in the cellular composition across different disease stages. This approach could be further exploited in cancer research and potentially serve as a companion diagnostic tool in tumor development, progression and during treatment.

## Simple Summary

Cervical biofluids collected during routine Pap smears contain a mixture of epithelial and immune cells that reflect cervical health and disease. This study demonstrates proof-of-concept that multicolor flow cytometry can be applied to these samples to identify and characterize cell populations in normal cervical samples as well as in different stages of cervical cancer. Our analysis identified major cell populations, including different types of epithelial cells and immune cells, and revealed that their composition within the samples changes with disease stage. We observed variability in both cellular composition and marker expression, highlighting the need for additional markers and larger patient cohorts. Nonetheless, this minimally invasive approach could potentially be used to monitor tumor cells, immune responses, and therapeutic outcomes over time.

## 1. Introduction

Cervical cancer remains the fourth most common cause of cancer-related death among women globally. It typically arises from cervical intraepithelial neoplasia (CIN) and progresses predominantly (~80%) as squamous cell carcinoma (SCC), with a smaller proportion (~20%) manifesting as adenocarcinoma [1]. Most cervical cancers originate in the transformation zone (TZ), a region of the cervix where columnar epithelium undergoes metaplasia into squamous epithelium, particularly during puberty. The meeting point of the different cell types on this interface, known as the squamocolumnar junction (SCJ) or transition zone, is especially vulnerable to high-risk human papillomavirus (HPV) infection and has a limited ability to mount effective immune responses [2,3].

Routine screening through Papanicolaou (Pap) smears or liquid-based cytology (LBC) relies on cytological evaluation of cells collected from the ecto- and endocervix. Over 95% of cervical malignancies are associated with persistent HPV infection [4], and molecular assays detecting HPV DNA, RNA, or circulating tumor DNA have been integrated into diagnostic workflows [5]. Despite these advances, the sensitivity of cytology remains highly variable, with false-negative rates averaging around 25%, even in well-established screening programs [6,7,8]. This variability is influenced by the quality of cervical sampling, particularly from the TZ and SCJ, and the subjective interpretation by cytopathologists.

To improve diagnostic consistency and objectivity, strategies enabling the profiling and quantification of specific cervical cell populations are needed. While cervical samples contain a heterogeneous mixture of epithelial and immune cells, many conventional techniques require purified populations to yield meaningful biomarker data. In contrast, flow cytometry uniquely allows for single-cell resolution and the analysis of mixed populations without physical separation, using gating strategies.

Multicolor flow cytometry has previously been explored for cervical cancer screening [9,10,11], primarily as a quality control measure for LBC samples. However, earlier efforts were limited by insufficient resolution in separating distinct cell populations, reliance on large sample volumes, and variable statistical robustness. Since then, advances in cytometry technology and marker panels have significantly improved resolution and sensitivity for complex biofluids.

In this study, we present a refined flow cytometry approach for characterizing cellular subpopulations in routine cervical swabs, with application across the spectrum of normal, precancerous, and cancerous lesions. We developed and applied a multiparameter staining strategy including both extracellular and intracellular markers to assess the cellular composition of cervical biofluids. This work serves as a proof-of-concept and technical foundation for future companion diagnostic tools in cervical cancer screening and monitoring.

## 2. Materials and Methods

### 2.1. Pap Smear Samples

Samples were provided by Universitätsklinikum Tulln. Acquisition as well as diagnosis was performed by an experienced pathologist according to the joint guideline of the OEGGG, AGO, AGK and OEGZ on the diagnosis and treatment of cervical intraepithelial neoplasia [12].

This study was approved by the Commission for Scientific Integrity and Ethics at the Karl Landsteiner Privatuniversität (ethic number 1008/2020) and conducted in accordance with the Declaration of Helsinki. All women gave written informed consent.

Details on patient data can be found in Table 1. Rovers Cervex-Brush Combi^®^ cell samplers (Rovers Medical Devices, Oss, The Netherlands, 380101031) were used for sampling. For the collection of pure endocervical samples, Cytobrush Plus^®^ cell collectors (Cooper Surgical, Trumbull, CT, USA, C0021), and for collection of pure ectocervical samples Cervex-Brush^®^ cell samplers (Rovers Medical Devices) were used. Simultaneous detection and identification of 28 HPV types (19 high-risk and 9 low-risk) was done during routine clinical diagnostics by Universitätsklinikum Tulln using real-time PCR Anyplex II HPV28 detection kit (Seegene, Seoul, Republic of Korea, HP7S00X). For transportation of samples 50 mL centrifuge tubes filled with 10 mL of DMEM (Dulbecco’s Modified Eagle’s Medium; Seegene, L0103-500) plus 1× Penicillin-Streptomycin (Euroclone, Pero, Italy, ECB3001D) were used. Transport was conducted on ice. Cervical biofluids received for flow cytometric analysis were numbered consecutively (Table 1).

### 2.2. Cell Harvest from Pap Smear Brushes

Pap smear brushes were transferred into a 6-well plate with 3 mL DMEM + 1× Penicillin-Streptomycin, which was placed on ice. Tissue and cells were detached from the brush by pipetting and/or with single-use tweezers. Small tissue fragments were manually separated by pipetting or by pulling it apart with tweezers. Cell and tissue suspension from 6-wells was transferred into the 50 mL tube again and empty wells were washed with 1 mL of DMEM + 1× Penicillin-Streptomycin. For dissolving the mucus 50 mM DTT (dithiothreitol; BDH, Singapore, 443852A) was added to the tube to get a final concentration of 5 mM DTT. The solutions were resuspended and the tube was rotated for 5 min at room temperature. After centrifugation with 600× *g* for 5 min the supernatant was discarded and the pellet was resuspended in 1 mL DMEM + 1× Penicillin-Streptomycin. To be able to count cells and lyse erythrocytes a 1:10 dilution with VersaLyse (Beckman Coulter, Singapore, A09777) was prepared where 3 µL of cells were added to 27 µL of VersaLyse and incubated for 10 min. Cell counting with a Neubauer counting chamber was followed by preparing samples for different experimental procedures.

### 2.3. Harvest of Cell Lines

Following cell lines from ATCC (Manassas, VA, USA) were used in this study: CaSki (HPV16+ cervical carcinoma), SiHa (HPV16+ squamous cell carcinoma), HeLa (HPV18+ cervical adenocarcinoma), U2OS (HPV- osteosarcoma) and A549 (HPV- lung carcinoma).

Cells were kept on ice during harvest whenever possible to avoid cell activation. Cells were washed twice with 1 mL of ice-cold 1× PBS (Biowest, Nuaillé, France, L0615-500) prior to trypsinization with 500 µL of 1× trypsin-EDTA (10X trypsin-EDTA (Sigma-Aldrich, St. Louis, MO, USA, 59418C) diluted with 1× PBS). Incubation was performed at 37 °C for 8–10 min, depending on how fast cells detached from the adhesive plastic surface. The reaction was stopped by adding 1 mL of RPMI 1640 (Biowest, L0498-500) + 10% FCS (fetal calf serum; Gibco, Waltham, MA, USA, 10270-106) or 1 mL of DMEM + 10% FCS. The cell suspension was transferred into precooled microcentrifuge tubes. After centrifugation at 600× *g* for 5 min at 4 °C, the cells were washed with 500 µL ice-cold 1× PBS (600× *g* for 5 min at 4 °C).

### 2.4. Flow Cytometry

Antibodies conjugated to fluorochromes were selected based on target antigen, expected expression levels, and spectral properties. All antibodies were purchased from validated commercial sources and, if applicable, used according to the manufacturer’s recommended dilution.

Cells were washed with 500 µL ice-cold 1× PBS (600× *g* for 5 min at 4 °C). Staining with fixable viability dye was conducted by adding “Zombie NIR™ Fixable Viability Kit” (Biolegend, San Diego, CA, USA, 423105) diluted 1:2000 in 1× PBS to the cell pellet. Zombie NIR was incubated for 15 min at 4 °C in the dark and cells were afterwards washed with 500 µL flow cytometry buffer (1× PBS + 2% BSA (bovine serum albumin, Sigma-Aldrich, A3294-100G)) at 600× *g*, for 5 min at 4 °C. Cells were fixed with 500 µL of FoxP3 fixation/permeabilization working solution (in compliance with the manufacturer’s protocol; Invitrogen, Waltham, MA, USA, 00-5523-00) for 30 min at 4 °C. Following a washing step (600× *g* for 5 min at 4 °C) with 500 µL of 1× permeabilization buffer (Invitrogen, 00-8333-56) the pellet was resuspended in 500 µL flow cytometry buffer (1× PBS + 2% BSA) and stored at 4 °C until staining.

This staining procedure was started by centrifugation at 600× *g* for 5 min at 4 °C of cells that were stored at 4 °C until staining. Cells were washed with 500 µL of 1× permeabilization buffer and the same centrifugation settings as before. Blocking was achieved by resuspending the pellet in 40 µL of 1× permeabilization buffer + 2% FCS and incubation for 15 min at room temperature. Without washing 10 µL of primary antibody (100X HPV E7 antibody mix (Valdospan, Tulln an der Donau, Austria, 206-004-1/206-068-1) 1:10 diluted in 1× permeabilization buffer + 2% FCS) were added prior to incubation for 30 min at room temperature in the dark. During this incubation time the microcentrifuge tubes were vortexed and centrifuged for about 1 s with a table centrifuge to ensure homogeneous mixture of the cell/antibody solution and avoid droplets on the inside of the tubes. Cells were washed with 500 µL of 1× permeabilization buffer at 600× *g* for 5 min at room temperature. With 50 µL of secondary antibody (1:125 diluted Anti-mouse IgG (H + L), F(ab’)2 Fragment, PE (Phycoerythrin) Conjugate (Cell Signaling, Danvers, MA, USA, 8887) in 1× permeabilization buffer) cells were incubated for 30 min at room temperature in the dark. Once again microcentrifuge tubes were vortexed and centrifuged for about 1 s with a table centrifuge during incubation. Two washing steps, with 500 µL of 1× permeabilization buffer each, were conducted at 600× *g* for 5 min at room temperature. For directly labeled antibody staining seven different master mixes (further named panels) were produced. 50 µL of directly labeled antibody master mix were added to the cells and incubated for 30 min at room temperature in the dark. Following directly labeled antibodies were used: p16^INK4a^ (Cell Signaling, 43161S), Ki-67 (Biolegend, 350526), EpCAM (Biolegend, 324218), CD45 (Biolegend, 304042) and Cytokeratin 8 (Thermo Fisher Scientific, Waltham, MA, USA, 11-9938-82). During incubation, the microcentrifuge tubes were vortexed and centrifuged for around 1 s with a table centrifuge. Cells were washed with 500 µL of 1× permeabilization buffer at 600× *g* for minutes at room temperature and resuspended in an appropriate volume (depending on the sample between 120 µL and 200 µL) of flow cytometry buffer. Antibodies were validated through isotype controls and FMO (fluorescence minus one) controls to establish gating boundaries. Furthermore, negative control samples were used, when available, to assess specificity (e.g., U2OS for p16^INK4a^, EpCAM).

To correct for spectral overlap between fluorochromes, single-stained compensation controls were prepared using compensation beads (AbC™ Total Antibody Compensation Beads (Invitrogen, A10513)). Beads were stained with the same antibody-fluorochrome conjugates used for cell staining, according to the manufacturer’s protocol. Compensation beads without antibody conjugate capture capacity served as negative control. Compensation matrices were generated automatically using CytExpert version 2.4.0.28 (Beckman Coulter) and verified manually to ensure accuracy. Supplementary Figure S6 shows compensation results for all separate channels (Supplementary Figure S6A) and the applied compensation matrix (Supplementary Figure S6B).

All measurements and analyses were performed using a four-laser CytoFLEX S flow cytometer (Beckman Coulter) at Connective Base GmbH (Vienna, Austria), using CytExpert version 2.4.0.28 for gating, Excel (Microsoft, Redmond, WA, USA) for calculations of ex-ported CytExpert values and GraphPad Prism 6 version 6.01 (GraphPad, San Diego, CA, USA) for producing and designing graphs. Median fluorescence intensity (MFI) was used throughout the study.

Gates with less than 50 events were excluded for analysis due to otherwise non-conclusive results in these gates. Samples with less than 5000 cells in the “cells” gate (which is the first gate to exclude obvious debris and bubbles) were also excluded from the analysis because nearly no events would remain in lower hierarchy gates.

### 2.5. Fluorescence Activated Cell Sorting

Cervical biofluid samples were sorted by fluorescence activated cell sorting (FACS) using a FACSAria Fusion cell sorter (BD, Franklin Lakes, NJ, USA) at Connective Base GmbH. Different characterized populations were sorted into microcentrifuge tubes containing DMEM + 10% FCS to prevent cells from drying out. Sorted cells were used for slide preparation and microscopic imaging. It was attempted to sort all populations used for flow cytometric analysis. However, the cell count of lower hierarchy gates was not high enough to sort them with this method. Therefore, only the size dependent gates (FSC/SSC) could be validated through FACS.

### 2.6. Pap Staining of Cervical Biofluids

Cells were centrifuged with 600× *g* for five minutes to concentrate on the bottom of a microcentrifuge tube. Around 10 to 20 µL were plated onto a microscopy slide, air dried and fixated in 96% ethanol (Carl Roth, Karlsruhe, Germany, T171.7) for 10 min. Slides with fixated Pap smear cells were rehydrated by incubation for 10 s with 96%, 80% 70%, 50% and double-distilled water. Then slides were stained with Papanicolaou’s solution 1a Harris’ hematoxylin solution (Merck, Singapore, 1.092530.500) for three minutes at room temperature. After washing with running tap water for three minutes, slides were incubated in 70%, 80% and 96% ethanol for 30 s each. Slides were stained with Papanicolaou’s solution 2a Orange G solution (Merck, 1.068880.500) for three minutes at room temperature. The next step was washing two times with 96% ethanol for 30 s. This was followed by staining in Papanicolaou’s solution 3b polychromatic solution EA50 (Merck, 1.09272.0500) for three minutes. Subsequently, slides were washed two times with 96% ethanol for 30 s. Five minutes of incubation with 100% ethanol and two times incubation with Roticlear (Carl Roth, A538.1) followed. Slides were mounted with Eukitt non-aqueous mounting media (Sigma-Aldrich, 03989-100 mL).

### 2.7. HE and IHC Stained Tissue Sections

Staining of cervical specimen for hematoxylin-eosin (HE) as well as immunohistochemistry (IHC) with antibodies against p16^INK4a^ and Ki-67 was organized and performed by Universitätsklinikum Tulln for routine clinical diagnosis. All HE slides were scanned using a Vectra Polaris slide scanner (with integrated Hamamatsu camera and 20× objective; Akoya Biosciences (Marlborough, MA, USA) whereas p16^INK4a^ and Ki-67 slides were scanned with a TissueFAX slide scanner (with integrated Baumer camera and 20× objective; TissueGnostics, Vienna, Austria).

### 2.8. Statistics

One- and two-way ANOVAs were performed using GraphPad Prim 6 version 6.01. To identify which specific groups differ from each other, Tukey’s multiple comparison was carried out. Significance levels are given with * for *p* ≤ 0.05, ** for *p* ≤ 0.01, *** for *p* ≤ 0.001 and **** for *p* ≤ 0.0001.

## 3. Results

**Figure 1:**
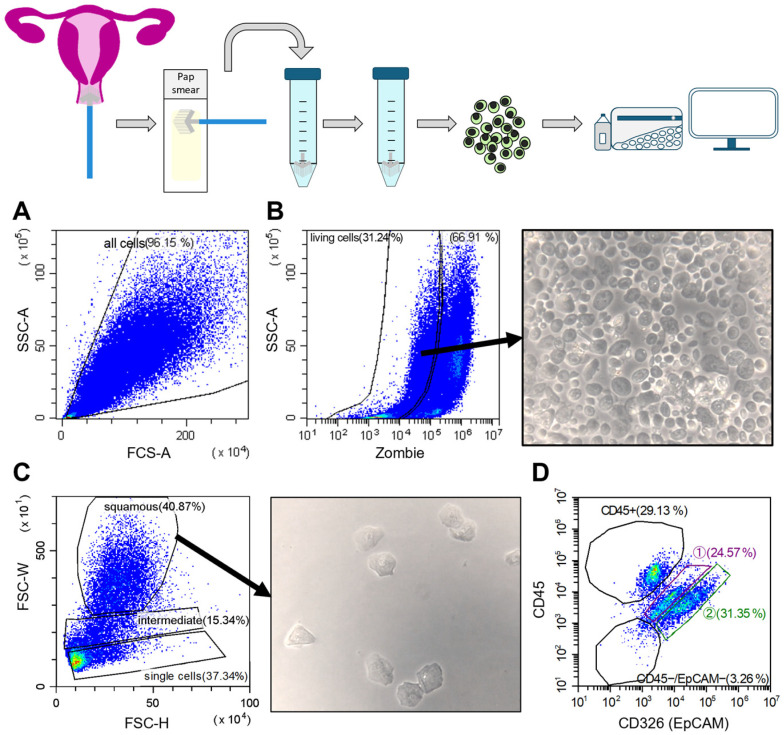
Schematic illustration of cervical biofluid preparation and flow cytometric gating strategy, exemplified by a healthy sample. The schematic (top) shows sample procurement from the cervix with a brush during gynecological examination, followed by the routine Pap smear on an objective slide. The same brush is then transferred into a tube containing transport medium. Cells are harvested from the brush, processed, and prepared for flow cytometric analysis. The gating strategy (below) is illustrated using an HPV negative Pap II cervical biofluid sample. (**A**) Forward scatter height (FSC-H) vs. side scatter (SSC) plot showing gating of “all cells” present in the cervical biofluid mixture. (**B**) Left: SSC vs. Zombie plot of “all cells” used to exclude dead cells (Zombie positive). Right: representative cell image (10× objective) of the “living cells” population obtained by fluorescence-activated cell sorting (FACS), showing the mixture of cell types and sizes present in cervical biofluid. (**C**) Left: FSC-W vs. FSC-H plot of “living cells” showing three main gates: “single cells” (lowest gate), “intermediate” (middle gate), and “squamous” cells (uppermost gate). The “squamous” gate was sorted by fluorescence-activated cell sorting (FACS), and a representative image was acquired under the microscope (10× objective). (**D**) CD45 vs. epithelial cell adhesion molecule (EpCAM) plot of “single cells,” revealing four populations. Cells in the “CD45+” gate represent immune cells. The lower left gate (CD45−/EpCAM−) was excluded from analysis, as it primarily consisted of debris, dead cells, or spillover events from other gates. Populations ① and ② were considered cervical epithelial cells.

Cervical biofluids obtained from routine Pap smear samples contain a heterogeneous mixture of cell populations. To investigate these populations, we developed a sample preparation method that allows both cytological assessment and multicolor flow cytometry from the same specimen (Figure 1). This required high recovery of viable cells from the brush while minimizing disruption of cell integrity, particularly important given the viscous and mucinous nature of cervical fluids. Several brush types were tested to optimize sample collection and cell viability (detailed in the Methods Section).

### 3.1. Flow Cytometric Analysis of Pap Smear Samples

First, HPV negative, noncancerous samples were analyzed as a reference for healthy tissue (Figure 1). Debris and dead cells were excluded using the Zombie (Fixable Viability Kit) (Figure 1A,B). Dead cells were mostly larger epithelial cells with high side and forward scatter (SSC, FSC) profiles. Microscopy confirmed a heterogeneous cell mixture in viable fractions (Figure 1B). The FSC-W versus FSC-H plot revealed three subpopulations: “squamous” (largest cells), “intermediate,” and “single cells” (smallest, dense cluster; Figure 1C). Squamous epithelial cells, identified in the uppermost gate, were confirmed via fluorescence-activated cell sorting (FACS) and microscopy as stratified squamous ectocervical cells.

The “intermediate” population, a mix of squamous and small cells, was not further analyzed due to its heterogeneity. The “single cells” population was further characterized via CD45 and EpCAM expression (Figure 1D).

Analysis of the “single cells” gate for the immune marker CD45 identified a group of immune cells within the cervical biofluid from normal tissue. While EpCAM has been reported as negative in squamous epithelial tissue of the cervix, it is known to be expressed in cervical intraepithelial lesions and cancerous cells [13,14,15]. However, an EpCAM positive cell population was readily detectable in normal cervical biofluid samples. This finding challenges the intended use of EpCAM as a negative marker in healthy cell populations, suggesting it may not be a reliable tumor-specific marker for identifying pathological biofluid samples. EpCAM positive cells detected in healthy samples were sorted into the “population 2” gate (Figure 1D). To further characterize the cells in “population 2”, biofluid Pap samples were collected separately from the endocervical and ectocervical regions of women with noncancerous cervical tissue. This approach aimed to better differentiate and localize the two main epithelial cell types of the cervix: columnar and squamous epithelium.

### 3.2. Endocervical Cell Characterization

**Figure 2:**
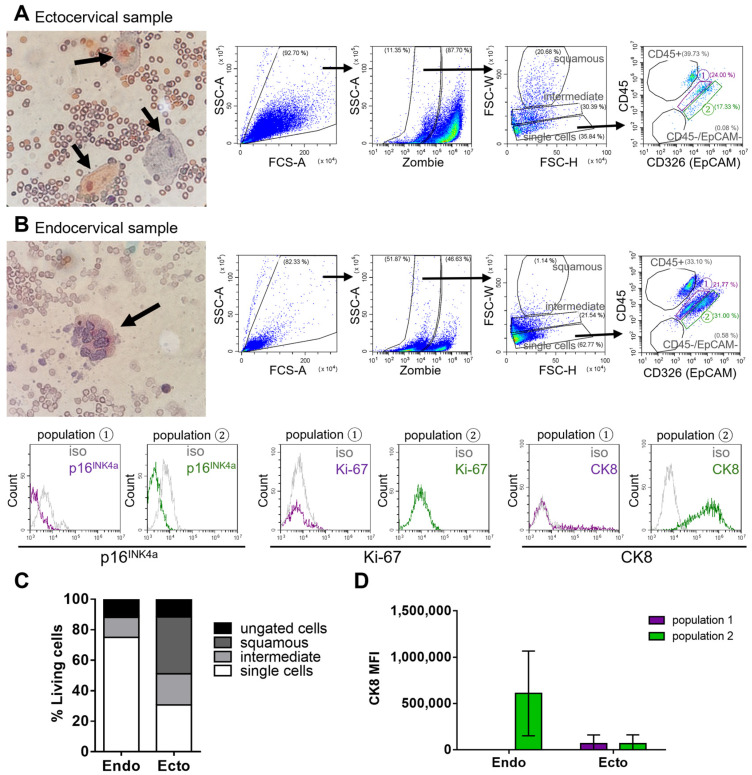
Analysis of cervical biofluids from ectocervical and endocervical regions obtained from the same woman. (**A**) Left: representative image (10× objective) of a native ectocervical biofluid sample, Pap-stained prior to fixation for flow cytometry, showing erythrocytes (small reddish cells/circles) and large squamous cells (arrows). Right: flow cytometric gating scheme of an ectocervical sample. FSC vs. SSC plot shows gating of “all cells”. SSC vs. Zombie (Fixable Viability Kit) plot of “all cells” was used to exclude dead cells (Zombie positive). FSC-W vs. FSC-H plot of “living cells” shows three gates: “single cells” (lowest gate), “intermediate” (middle gate), and “squamous” cells (uppermost gate). CD45 (immune cells) vs. epithelial cell adhesion molecule (EpCAM) plot of “single cells” reveals four populations, with populations ① and ② of particular interest for distinguishing ecto- and endocervical cells. (**B**) Left: representative image (10× objective) of a native endocervical biofluid sample, Pap-stained prior to fixation for flow cytometry, showing erythrocytes (small reddish cells/circles) and a cluster of columnar endocervical cells (arrow). Right: flow cytometric gating scheme of an endocervical sample, with gates as shown in (**A**). Below: representative histograms of p16^INK4a^, Ki-67, and CK8 expression in population ① (purple) and population ② (green) compared to isotype (iso; grey). (**C**) Quantification of flow cytometric analysis of four samples (two women, each with one ectocervical and one endocervical biofluid analyzed) based on FSC-W vs. FSC-H distribution of cells in ecto- and endocervical biofluids. (**D**) Quantification of CK8 expression in the same four samples, analyzed separately in population ① and population ②.

To further investigate the origin of EpCAM+ cells in healthy samples, biofluids obtained specifically from the endocervical and ectocervical regions were analyzed. As expected, ectocervical samples predominantly contained large squamous cells identified by their size and distinct light scattering properties in the FSC-W parameter, as previously shown (Figure 2A,C).

In contrast, endocervical cells are smaller and require additional evaluation to distinguish them from other cells in cervical biofluids. Analysis of only the endocervical region, using the established flow cytometry gating strategy, identified the EpCAM positive “population 2” as uniquely present in the endocervical sample of normal tissue (Figure 2B). This suggests that these EpCAM positive cells represent the columnar epithelial cells of the endocervix. Supporting this, the cells in “population 2” also expressed cytokeratin 8 (CK8), a marker known to be present in endocervical cells (Figure 2B,D) [13]. These findings suggest that CK8 expression can help distinguish endocervical from ectocervical epithelial cells.

Overall, biofluid samples from the endocervical region contained a higher number of single cells, whereas ectocervical samples were predominantly composed of squamous epithelial cells. Due to the distinct size and light-scattering properties of squamous cells, and the minimal presence of other cell types, no further staining was conducted on the ectocervical samples.

Notably, in noncancerous endocervical samples, neither p16^INK4a^ nor Ki-67, both established markers of abnormal cell proliferation, were detected.

### 3.3. Flow Cytometric Analysis of Cancer Cell Lines

After establishing and validating a flow cytometric gating strategy for the analysis of healthy cervical biofluids, the next objective was to determine the positioning of tumor cells within this established scheme. To differentiate normal cervical cells from tumor cells, the same gating approach applied to healthy cervical biofluid samples was used to analyze isolated populations from various tumor cell lines.

**Figure 3:**
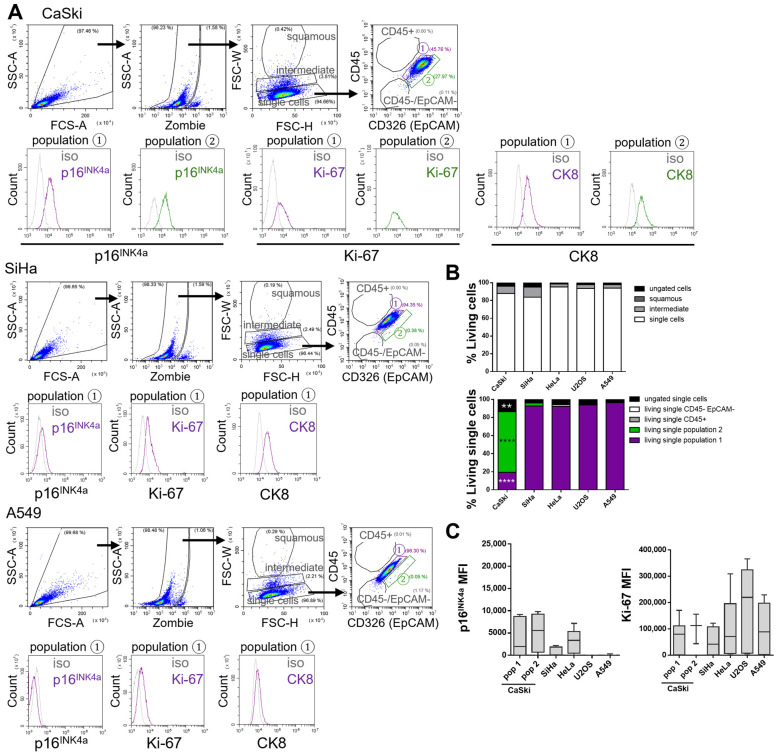
Flow cytometric analysis of epithelial cancer cell lines. (**A**) Dot plots and histograms of flow cytometry measurements showing an exemplary gating of CaSki (cervical SCC), SiHa (cervical SCC) and A549 (non-small cell lung cancer) cell lines. From left to right, FSC vs. SSC plot shows gating including “all cells”. From “all cells”, SSC vs. Zombie (Fixable Viability Kit) plot shows the exclusion of dead cells (Zombie positive). “Living cells” were plotted for FSC-W (Width) vs. FSC-H (Height) showing the gate for “single cells” (lowest gate). “Single cells” are further gated for CD45 vs. EpCAM to gate for immune cells and epithelial (cancer) cells, respectively. Histograms below show p16^INK4a^ and Ki-67 expression of population (pop) ① (left, purple) and for CaSki also population 2 (right, green) compared to isotype (iso; grey). For the other cell lines, no histograms for p16^INK4a^ nor Ki-67 of population ② (green) are shown due to the sparce number of cells in this gate. (**B**) Quantification of “living cells” in CaSki, SiHa, HeLa, U2OS, and A549 based on the FSC-W vs. FSC-H plot, gated for squamous (not present in the cell lines), intermediate, and single cells populations. The quantification shown below represents cell populations of the CD45 vs. EpCAM plot. The resulting sub-populations include CD45−/EpCAM−, CD45+ (not present in the cell lines), population ① and population ②, as indicated. mean of n = 6. Significance: CaSki vs. other cell lines *p* ≤ 0.01 (**), *p* ≤ 0.0001 (****) (**C**) Median fluorescence intensity (MFI) quantification of p16^INK4a^ and Ki-67 expression of population ① from various cancer cell lines (for CaSki also population ②). Values for U2OS p16^INK4a^ are below the detection threshold. median (min/max) of n = 5, (CaSki Ki-67 population ② n = 3).

Despite the availability of established cervical cancer cell lines, the correlation between marker expression in cultured tumor cells and corresponding patient-derived specimens remains limited in the literature. To characterize tumor cell populations for future application in clinical cervical biofluid samples, a panel of both HPV positive and HPV negative tumor cell lines was analyzed to determine cell size distribution and localization within the gating framework. The panel included the HPV16 positive cervical squamous cell carcinoma cell lines CaSki and SiHa, the HPV18 positive cervical adenocarcinoma cell line HeLa, the HPV negative lung adenocarcinoma cell line A549 (as a non-cervical epithelial control), and the osteosarcoma cell line U2OS (representing a distinct tissue type). Flow cytometry was used to analyze all cell lines (Figure 3; HeLa and U2OS shown in Supplementary Figure S1A).

Cells were first gated for live, single cells using FSC-H versus FSC-W parameters, with dead cells excluded by staining with the Zombie (Fixable Viability Kit). The majority of cells localized to the “single cells” gate, while cell doublets were found in the higher “intermediate” gate and were excluded from further analysis. This exclusion was critical, as only single cells can be reliably assessed for surface and intracellular marker expression by flow cytometry. Unlike cervical biofluid samples, cells from tumor cell lines localized exclusively to the “single cells” gate (Figure 3A and Supplementary Figure S1A).

Subsequent analysis of cell surface markers EpCAM (tumor cell marker) and CD45 (immune cell marker) revealed that all tumor cell lines lacked CD45 expression, as expected in pure tumor cell cultures. Most tumor cell lines localized to the “population 1” gate, with the exception of CaSki cells, which were predominantly found in “population 2”—a gate defined as EpCAM positive based on isotype controls (Supplementary Figure S2) [14]. Tumor cells were consistently restricted to “population 1” and “population 2.” This was further supported by intracellular staining for p16^INK4a^, a well-established surrogate marker in immunohistochemistry [16], and Ki-67, a proliferation marker [17] (Figure 3A,C and Supplementary Figure S1A).

As anticipated, the HPV positive cell lines CaSki, SiHa, and HeLa expressed p16^INK4a^, whereas the HPV negative lines A549 and U2OS were negative. All cell lines were positive for Ki-67, confirming their proliferative state. These findings validate the suitability of the established gating strategy for both surface and intracellular marker analysis in cervical biofluid samples.

In healthy cervical biofluids, CK8 was identified as a marker of endocervical cells located in “population 2.” In contrast, cervical tumor cell lines demonstrated high CK8 expression in both “population 1” and “population 2” (Figure 3A and Supplementary Figure S1A,B). Notably, CK8 expression was absent in “population 1” of healthy cervical samples (Figure 2B,D). These observations suggest that in patient-derived cervical biofluids, CK8 positive tumor cells may be identifiable in both “population 1” and “population 2,” whereas “population 2” may also contain normal endocervical epithelial cells.

### 3.4. Tumor Cell Identification in Patient-Derived Biofluidic Cervical Pap Samples

**Figure 4:**
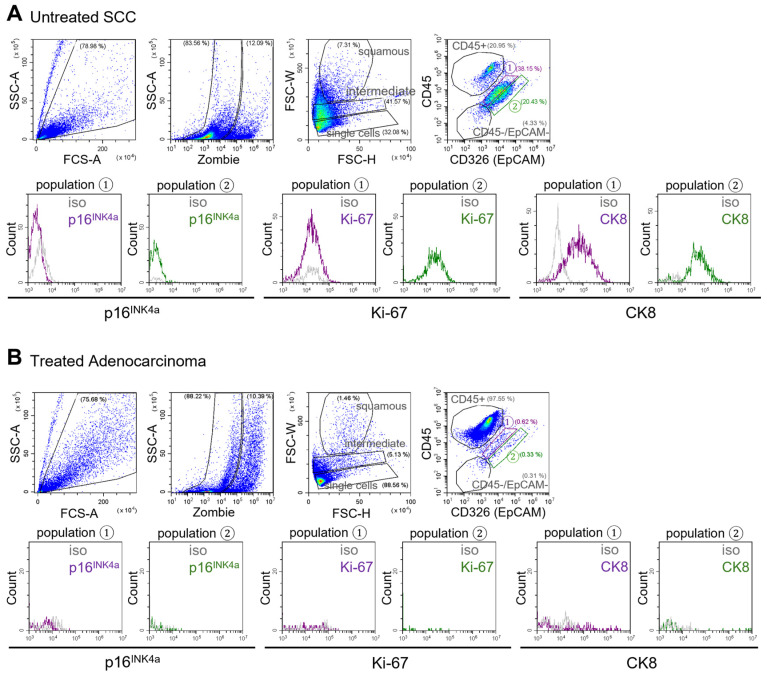
Flow cytometric analysis of Pap smear samples from cervical cancer patients. From left to right: FSC vs. SSC plot showing gating of “all cells”; SSC vs. Zombie (Fixable Viability Kit) plot of “all cells” showing exclusion of dead cells (Zombie positive); FSC-W (width) vs. FSC-H (height) plot of “living cells” with gates for “single cells” (lowest gate), “intermediate” (middle gate), and “squamous” cells (uppermost gate); and CD45 vs. EpCAM plot of “single cells” used to distinguish immune cells (CD45+) from epithelial (cancer) cells (EpCAM+). Histograms below display p16^INK4a^, Ki-67, and CK8 expression in population ① (left, purple) and population ② (right, green) compared to isotype (iso; grey). (**A**) Pap smear sample from a 45-year-old woman with untreated squamous cell carcinoma (SCC; PAP27). (**B**) Pap smear sample from a 38-year-old woman with chemotherapy-treated cervical adenocarcinoma (PAP28).

The established flow cytometry gating strategy, based on the analysis of cancer cell lines and healthy cervical biofluids, suggests that tumor-derived epithelial cells localize within both “population 1” and “population 2.” However, CK8 expression serves as a distinguishing feature, as CK8 positive cells within “population 1” are indicative of tumor origin, given that this population is CK8 negative in healthy samples (as shown above). To validate the presence of tumor cells within this population in clinical samples, cervical biofluids were collected from patients with cervical tumors of various histological subtypes, disease stages, and treatment backgrounds (Figure 4).

The same flow cytometry gating strategy established for normal cervical biofluids and tumor cell lines was applied to cervical biofluids collected from patients with cervical cancer. Particular attention was given to “population 1,” where tumor cells are anticipated to localize. Indeed, within this gate, a distinct CK8 positive cell population was identified in a sample from a patient with untreated squamous cell carcinoma (SCC; Figure 4A).

In contrast, cervical biofluid from a patient with a chemotherapy treated adenocarcinoma exhibited markedly fewer CK8 positive cells in “population 1,” with immune cells predominating in the sample (Figure 4B). Conversely, cervical biofluid from a patient with chemo-radiotherapy–treated SCC still contained few CK8 positive cells within “population 1” (Supplementary Figure S3A). However, in a separate case involving a patient with a large (7 cm), untreated SCC, the corresponding cervical biofluid showed a minimal presence of CK8 positive cells in the “population 1” gate (Supplementary Figure S3B).

These findings demonstrate that the established flow cytometry approach is applicable to patient-derived cervical biofluids and enables the identification of healthy endo- and ectocervical epithelial cells, tumor cells, and immune cell populations. They provide proof-of-concept for detecting tumor-associated markers in cervical biofluids but also highlight variability between samples, underscoring the need for larger, longitudinal studies. To address this inter-patient variability and gain a broader understanding of cellular and immunological changes during cervical disease progression, cervical biofluids from patients with a range of cervical pathologies and cancer stages were analyzed.

### 3.5. Cervical Biofluid Sample Characterization Corresponding to Disease Stage

**Figure 5:**
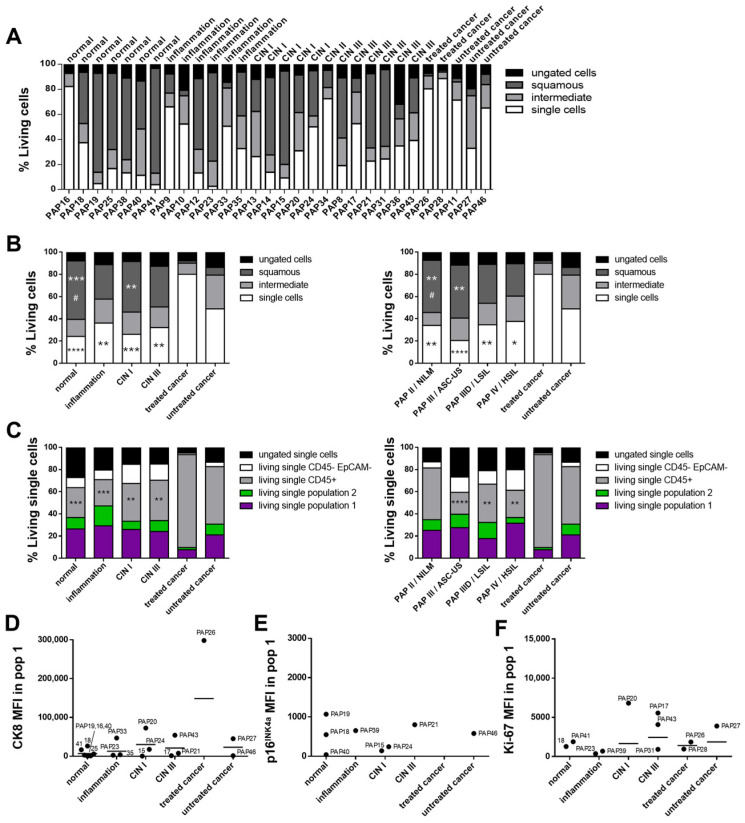
Evaluation of 30 Pap smear samples from normal cervical tissue to various stages of cervical neoplasia. (**A**) Individual Pap smear samples sorted according to disease stage. For each sample, the “squamous,” “intermediate,” and “single cell” populations were gated based on FSC-H vs. FSC-W. Samples are numbered chronologically according to the time of receipt. (**B**) Quantification of the “squamous,” “intermediate,” and “single cell” populations from the FSC-H vs. FSC-W plots, averaged across all Pap samples and grouped by CIN (**left**) or Pap/Bethesda (**right**) classification. Data represent the averages of samples shown in (**A**). (**C**) Quantification of cells from the CD45 vs. EpCAM plots, averaged across all Pap samples and grouped according to CIN (**left**) or Pap/Bethesda (**right**) classification. Data represent the averages of samples shown in Supplementary Figure S4A. (**D**–**F**) Samples with expression levels above isotype controls for CK8 (**D**), p16^INK4a^ (**E**), or Ki-67 (**F**) in population ① (pop 1) are shown, grouped by disease stage. Significance: vs. treated cancer, *p* ≤ 0.05 (*), *p* ≤ 0.01 (**), *p* ≤ 0.001 (***), *p* ≤ 0.0001 (****); vs. untreated cancer, *p* ≤ 0.05 (#).

With the major cell populations within cervical biofluids identified, the next step involved analyzing patient samples representing a range of disease stages, from normal epithelium and cervical intraepithelial neoplasia (CIN) to invasive cancers such as SCC, using multicolor flow cytometry (Figure 5 and Supplementary Figure S4). Individual patient results are presented in Figure 5A and Supplementary Figure S4A to illustrate variability between samples within the same disease stage.

Cervical biofluid samples were collected from patients with normal, inflammatory, and cancerous cervical tissues, as well as from those diagnosed with CIN I and CIN III (Table 1). CIN II was underrepresented, with only a single patient sample, and was therefore excluded from quantitative and statistical analyses. Among the five biofluid samples from cancer-bearing tissues, two were obtained from patients who had undergone therapeutic interventions, including systemic chemotherapy and/or radiotherapy.

Given that cervical samples are routinely classified, samples were grouped according to disease stage based on the cytological Pap or Bethesda classification as well as the histological CIN grading with an additional category for inflammatory tissues. For statistical analysis, cancer samples were further subdivided into treated and untreated groups.

Statistical analysis of cervical cancer samples, stratified into untreated and treated groups, revealed significant differences in the “single cells” population based on CIN classification. Treated cancer samples showed a marked increase in the proportion of “single cells” compared to samples classified as normal (*p* ≤ 0.0001), inflammatory (*p* ≤ 0.01), CIN I (*p* ≤ 0.001), and CIN III (*p* ≤ 0.01; Figure 5B, white bars). Conversely, the proportion of squamous cells was significantly reduced in treated cancer samples compared to normal (*p* ≤ 0.001) and CIN I (*p* ≤ 0.01) samples (Figure 5B, dark gray bars). Similarly, untreated cancer samples also had significantly fewer squamous cells than normal samples (*p* ≤ 0.05).

Comparable trends were observed when samples were grouped according to Pap/Bethesda classification. The “single cells” population was significantly increased in treated samples compared to Pap II/NILM (*p* ≤ 0.01), Pap III/ASC-US (*p* ≤ 0.0001), Pap IIID/LSIL (*p* ≤ 0.01), and Pap IV/HSIL (*p* ≤ 0.05) samples (Figure 5B, white bars). In parallel, the squamous cell population was significantly reduced in treated samples compared to Pap II/NILM and Pap III/ASC-US (both *p* ≤ 0.01). Untreated cancers also showed a lower squamous cell proportion compared to Pap II/NILM samples (*p* ≤ 0.05; Figure 5B, dark gray bars).

Immune cells, identified by CD45 expression, were significantly elevated in treated cancer samples compared to normal (*p* ≤ 0.001), inflammatory (*p* ≤ 0.001), CIN I (*p* ≤ 0.01), and CIN III (*p* ≤ 0.01) tissues (Figure 5C). A similar pattern was observed using Pap or Bethesda classification: treated cancer samples exhibited significantly higher immune cell levels than Pap III/ASC-US (*p* ≤ 0.0001), Pap IIID/LSIL (*p* ≤ 0.01), and Pap IV/HSIL (*p* ≤ 0.01) samples.

Patients were also stratified based on their HPV subtype identified via qPCR. Only HPV16 positive samples exhibited significantly fewer squamous cells (Supplementary Figure S4D,E). However, this finding should be interpreted with caution, as there were only three samples in this group, one of which was a tumor.

CK8 expression was used as a potential marker to identify tumor cells within individual biofluid samples. Analysis of median fluorescence intensity (MFI) for CK8 within “population 1” across all disease stages revealed that many samples displayed low or borderline positivity after subtraction of the isotype control (Figure 5D). In about half of the analyzed samples, CK8 expression was slightly elevated, but no clearly defined CK8 positive population could be identified within the gating, suggesting that isotype subtraction alone may not provide sufficient sensitivity, and a more stringent threshold may be required.

A subset of samples did, however, show distinct CK8 positive populations and were examined in greater detail. For instance, sample “PAP33” contained only a few cells in “population 1” and some scattered cells in “population 2,” potentially skewing CK8 results. Sample “PAP20” also demonstrated dysplastic cells on Pap smear analysis. “PAP43” was categorized as Pap IV and displayed extensive lesions with dysplastic cells. Interestingly, sample “PAP18,” which was classified as normal tissue, contained a CK8 positive population within “population 1,” warranting further investigation.

Analysis of p16^INK4a^ expression in “population 1” showed that only a few samples had fluorescence intensities above the isotype control (Figure 5E; “population 2” in Supplementary Figure S4B). These levels were insufficient to reliably distinguish tumor cells or determine HPV status in individual samples. To further explore this, immunohistochemically (IHC) stained tissue sections were obtained from patients in the cohort who underwent surgical resection (Supplementary Figure S5). However, IHC analysis also revealed inconsistencies: some HPV positive tissues identified via routine PCR showed p16^INK4a^ negative abnormal epithelial cells, indicating that p16^INK4a^ alone may not reliably reflect HPV status in clinical samples.

Ki-67 MFI within “population 1” indicated positive expression in several samples across different disease stages (Figure 5F; “population 2” in Supplementary Figure S4C). However, this marker alone was insufficient to classify individual samples as tumor positive. IHC staining revealed that Ki-67 positive cells were detectable in the basal cell area of unaffected epithelium, while in abnormal tissue, areas of Ki-67 positive cells are interspersed through the tissue hierarchy, also some in close proximity to the outermost cell area (Supplementary Figure S5).

## 4. Discussion

In this study, we provide proof-of-concept that multicolor flow cytometry can be applied to analyze cervical biofluids in a minimally invasive manner, enabling the identification and characterization of epithelial and immune cell populations across the spectrum of cervical health and disease. By applying a consistent gating strategy, we distinguished between squamous and columnar epithelial cells, identified immune cells, and explored tumor-associated marker expression in both healthy and cancer-derived cervical samples.

Primary cervical tumors predominantly grow in proximity to the squamocolumnar junction. To facilitate differentiation of the cells that normally populate these regions, biofluids were retrieved from the inner part (endocervix) as well as from the outer part (ectocervix) of noncancerous cervical tissues during routine Pap examinations. The intermediate and superficial epithelial squamous cells represent the largest cells isolated from cervical biofluids. Most of them were identified as dead at the time of staining and therefore excluded early during the analysis procedure. These cells may be damaged by shearing forces during pipetting due to their large size of more than 1000 µm. Regardless, due to the technical setup of flow cytometers, these cells are too large for proper multicolor flow cytometric analysis and were only identified based on their light-scattering properties in FSC and SSC.

Other cells present in the cervical biofluid mixture require further differentiation based on the expression of specific surface markers. Based on prior immunohistochemical studies showing that cytokeratin 8 (CK8) is expressed in endocervical but not in ectocervical cells of cervical tissue [13,18], this marker was used to identify the presence of endocervical cells with flow cytometry. We highlight CK8 as an important discriminator between endocervical and ectocervical cells not only in tissue sections but also in biofluids analyzed with flow cytometry. Tumor cells could also be identified as CK8 positive, confirming early immunohistochemical findings [19,20]. In line with our biofluid data, immunohistochemical staining of tissue sections revealed that ectocervical squamous cells do not contain cytokeratin 8 [21,22]. EpCAM, similarly, is absent on normal squamous epithelia but shows increased expression during progression from CIN I to CIN III and is detectable in some squamous carcinomas [13]. Expression of EpCAM in cervical tumor cells was validated using tumor cell lines, though not all epithelial cancer cell lines express it. CaSki cells in particular demonstrated higher EpCAM expression compared to other lines, consistent with prior comparisons of CaSki, SiHa, and HeLa cells [23]. We also observed EpCAM expression on patient-derived healthy endocervical cells, confirming that EpCAM is not tumor-specific [13].

Nevertheless, our gating strategy allowed partial discrimination between endocervical and tumor-derived EpCAM+ cells based on expression intensity. Richter et al. reported EpCAM overexpression in aggressive cervical primary cell lines [24], raising the possibility that some tumors may display stronger EpCAM expression than observed here. To clarify these patterns, additional tumor samples need to be analyzed.

We also sought to evaluate classical markers p16^INK4a^ and Ki-67, widely used in cervical cancer diagnostics as dual staining [25] or individual [26]. These markers performed as expected in tumor cell lines but were not consistently informative in patient-derived biofluids. While Ki-67 is commonly used as an adjunct marker to improve diagnostic accuracy when morphology alone is insufficient [27], its cell-cycle–dependent variability complicates interpretation. In cervical tissues, Ki-67 upregulation of scattered cells was observed with immunohistochemical staining. It shows that patient-derived tumors do not consist of only one homogenous tumor cell population, but the cells vary in their expression of cellular markers. Maximal expression is restricted to mitotic cells, while late G1 and early S phase show only slightly elevated expression [28,29]. This suggests that not all Ki-67 negative cells are resting cells, because some late G1 cells cannot be distinguished from non-cycling cells. Even in homogenous tumor cell lines, Ki-67 expression differs markedly across cells, explaining its limited discriminatory power in cervical biofluids. Although not useful for tumor cell identification, Ki-67 may still provide information on tumor aggressiveness and treatment responsiveness, as pre-treatment Ki-67 levels have been correlated with therapy outcomes [30].

Similarly, p16^INK4a^ is associated with HPV positive tumors [31], but p16^INK4a^ negative HPV positive tumors are well documented [32]. Large flow cytometric analyses have also shown minimal differences between HPV positive and HPV negative women, suggesting limited diagnostic value in small cohorts or individual patients [33]. Our immunohistochemical analysis confirmed that p16^INK4a^ does not reliably distinguish HPV positive from HPV negative tumors. Accordingly, the use of p16^INK4a^ and Ki-67 in cervical biofluids must be interpreted with caution, as neither marker consistently identified tumor cells. Likewise, their combined use does not achieve reliable classification in individual patients [34].

Among all tested markers, CK8 emerged as the most promising discriminator for tumor cells in cervical biofluids. However, one normal cervical biofluid sample displayed a CK8+ population despite clinical follow-up confirming the absence of malignancy. Without additional samples from this patient, we can only speculate whether this represented a one-time effect, shedding of endometrial cells, or a subpopulation of basal/parabasal squamous cells. Indeed, earlier immunohistochemical work indicated weak CK8 expression in basal ectocervical cells [20]. Due to limitations in the fluorochrome panel and cell numbers per sample, we did not include CK7, CK5/p63, or MUC1 in this pilot study. Their inclusion in future studies would strengthen discrimination between basal and glandular epithelial subsets. In contrast, one squamous cell carcinoma sample displayed no CK8+ population, consistent with reports that not all SCCs are CK8 positive [20]. These findings highlight variability across patient-derived samples, reinforcing that our results represent proof-of-concept rather than diagnostic certainty. More tumor samples must be analyzed to establish the robustness of CK8 as a marker.

In addition to epithelial characterization, we examined cellular composition across disease stages. Cancer biofluids showed fewer squamous cells and more single cells compared to healthy samples, reflecting the disrupted tissue organization in tumors. Immune cells were readily distinguished by CD45. Interestingly, their quantity was not markedly increased in patients with signs of cervical inflammation, potentially due to focal inflammatory sites being underrepresented in bulk sampling. Nonetheless, tumor biofluids revealed variable immune infiltration, with some untreated tumors showing high immune content and others similar to healthy samples. Prior studies suggest increased T-cell infiltration in cervical cancer [35], but larger sample sets are required to validate immune monitoring by biofluids.

Our approach could be readily expanded to include immune subtypes, enabling more detailed immunophenotyping. Prior work using transcriptomic profiling of cervical cancer has highlighted prognostic and therapeutic roles of immune subsets [36,37]. Similarly, Punt et al. flow-sorted cervical tumor epithelial and immune cells to identify biomarkers via RNA seq [37]. Following up such approaches with biofluid cytometry could allow minimally invasive immune profiling. Importantly, treated cancer samples in our study displayed elevated immune fractions, suggesting possible therapy-induced immune responses. This implies that cervical biofluid analysis could also be useful for monitoring immune responses to therapy [38,39]. As immune dynamics are typically assessed only through invasive biopsy, biofluid analysis could offer a practical, non-invasive alternative for repeated sampling throughout treatment. Beyond cervical cancer, this methodology could potentially be applied to related gynecological malignancies. By using different sampling brushes, biofluid collection could be extended to endometrial tissues, broadening its application to endometrial neoplasia [40].

This method’s strength lies in its minimal invasiveness, feasibility for repeated sampling, and potential utility for companion diagnostics and therapy monitoring. However, our study was exploratory, and the sample size was limited. A larger cohort study is required to validate these preliminary observations, refine gating strategies, and assess the diagnostic utility of individual markers.

## 5. Conclusions

Herein, we identified the major cell populations in cervical biofluid samples and established a gating strategy suitable for individual sample analysis. Using multicolor flow cytometry, we demonstrated proof-of-concept for distinguishing squamous and columnar epithelial cells, immune cells, and potential tumor-associated cells across different stages of cervical health and disease. Treated cancer samples in particular displayed a distinct pattern, characterized by a reduction in squamous epithelial cells and an increase in small, single cells, with immune cells being most prominently elevated.

This approach highlights the potential of cervical biofluids as a minimally invasive and repeatable source for both basic biological insights and translational applications. The method not only enables the study of epithelial and tumor-associated markers but also allows assessment of immune cell dynamics, which could be valuable for monitoring therapeutic responses over time. Thus, cervical biofluid analysis by flow cytometry offers an attractive prospective tool for companion diagnostics and therapy monitoring.

Among the markers examined, CK8 showed promise as a discriminator for tumor cells, while EpCAM, p16^INK4a^, and Ki-67 yielded variable or inconsistent results. We emphasize that these findings are illustrative of potential patterns rather than conclusive evidence for diagnostic use. Larger patient cohorts and expanded marker panels are required to validate marker specificity, improve resolution of cellular subsets, and assess robustness across clinical settings.

In summary, this work establishes the feasibility of multicolor flow cytometry for cervical biofluids, demonstrating proof-of-concept but not yet clinical applicability. The observed variability underscores the need for additional tumor samples, longitudinal studies, and further marker development to enable reliable discrimination of cell populations in individual patients.

## Figures and Tables

**Table 1:** Patient characteristics.

Sample ID	Age [Years]	Pap Grade	CIN Grade	Bethesda System *	Inflammation	Tumor	HPV Status/Group	HPV Type	Type of Smear	Comment	Summary					
PAP8	30	IV	CIN III	HSIL	-	-	non HPV16/18 hr	33	endo- & ecto	-			**Amount**			**Amount**
PAP9	58	IV	no CIN	HSIL	chronic cervicitis	-	non HPV16/18 hr	56	endo- & ecto	-	**Age range**			**HPV type**		
PAP10	24	III D	no CIN	LSIL	cervicitis	-	HPV16 and non HPV16/18 hr	16, 66, 42	endo- & ecto	-		<20	0		7	1
PAP11	81	III	-	ASC-US	-	SCC	non HPV16/18 hr	66	endo- & ecto	untreated		20–30	6		16	5
PAP12	40	III	no CIN	ASC-US	inflammation	-	non HPV16/18 hr	31	endo- & ecto	-		31–40	9		18	3
PAP13	54	IV	CIN I	HSIL	-	-	HPV16	16, 43	endo- & ecto	-		41–50	12		26	1
PAP14	43	III D	CIN I	LSIL	-	-	non HPV16/18 hr	53	endo- & ecto	-		51–60	7		31	5
PAP15	48	III D	CIN I	LSIL	-	-	non HPV16/18 hr	35	endo- & ecto	-		61–70	0		33	2
PAP16	27	II	no CIN	NILM	-	-	negative	-	endo- & ecto	-		>70	1		35	1
PAP17	42	III	CIN III	ASC-US	-	-	non HPV16/18 hr	26, 53, 7, 73, 82	endo- & ecto	-					42	1
PAP18	28	II	no CIN	NILM	-	-	negative	-	endo- & ecto	-	**Pap grade**				43	1
PAP19	45	II	no CIN	NILM	-	-	negative	-	endo- & ecto	-		I	0		51	1
PAP20	56	III D	CIN I	LSIL	-	-	non HPV16/18 hr	53, 66, 82	endo- & ecto	-		II	7		52	2
PAP21	32	III D	CIN III	LSIL	-	-	HPV18	18	endo- & ecto	-		II/III	1		53	4
PAP22	31	III G	CIN II	AGC	-	-	non HPV16/18 hr	31	endo- & ecto	only IHC		III	9		56	3
PAP23	58	III	-	ASC-US	chronic cervicitis	-	negative	-	endo- & ecto	-		III D	9		58	2
PAP24	29	II	CIN I	NILM	-	-	non HPV16/18 hr	31, 33	endo- & ecto	-		III G	1		59	1
PAP25	49	II	no CIN	NILM	-	-	negative	-	endo- & ecto	-		IV	4		66	3
PAP26	53	no info	-	SSC	-	SCC	non HPV16/18 hr	hr	endo- & ecto	treated		V	0		73	1
PAP27	55	no info	-	SSC	-	SCC	HPV16	16	endo- & ecto	untreated		no info	4		82	3
PAP28	39	no info	-	adenocarcinoma	-	adenocarcinoma	non HPV16/18 hr	hr	endo- & ecto	treated					negative	8
PAP31	32	III	CIN III	ASC-US	-	-	negative	-	endo- & ecto	-	**CIN**				no info	2
PAP33	36	III D	-	LSIL	cervicitis	-	HPV16 and non HPV16/18 hr	16, 53, 56	endo- & ecto	-		no CIN	10			
PAP34	39	III D	CIN II	LSIL	-	-	HPV16 and non HPV16/18 hr	16, 51, 52, 58, 82	endo- & ecto	-		I	6	HPV group		
PAP35	46	II/III	-	NILM/ASC-US	endocervictis	-	negative	-	endo- & ecto	-		II	2		HPV16	5
PAP36	35	III	CIN III	ASC-US	-	-	HPV18	18	endo- & ecto	-		III	7		HPV18	3
PAP38	45	II	no CIN	NILM	-	-	non HPV16/18 hr	56	endo- & ecto	-		no info	10		non HPV16/18 hr	21
PAP39	56	III D	-	LSIL	inflammation	-	HPV18	18	endo- & ecto	atrophy					negative	9
PAP40	34	III	-	ASC-US	-	-	non HPV16/18 hr	52	endo- & ecto	-	**Inflammation**					
PAP41	47	III	no CIN	ASC-US	-	-	non HPV16/18 hr	31, 59	endo- & ecto	-		total	9			
PAP43	43	IV	CIN III	HSIL	-	-	non HPV16/18 hr	31	endo- & ecto	-						
PAP44	55	III	CIN I	ASC-US	inflammation	-	negative	-	endo only	same patient as PAP45	**Tumor**					
PAP45	55	III	CIN I	ASC-US	inflammation	-	negative	-	ecto only	same patient as PAP44		total	5			
PAP46	46	no info	-	SSC	-	SCC	negative	-	endo- & ecto	untreated		SCC	4			
PAP49	49	II	-	NILM	chronic cervicitis	-	negative	-	ecto only	same patient as PAP50		Adeno	1			
PAP50	49	II	-	NILM	chronic cervicitis	-	negative	-	endo only	same patient as PAP49		Untreated	3			
PAP53	24	III D	CIN III	LSIL	-	-	non HPV16/18 hr	58	ecto only	same patient as PAP54		Treated	2			
PAP54	24	III D	CIN III	LSIL	-	-	non HPV16/18 hr	58	endo only	same patient as PAP53						

* translated from Pap grade according to Reich et al, 2018 [12]. NILM = negative for intraepithelial lesion or malignancy, ASC-US = atypical squamous cells–undetermined significance, LSIL = low grade squamous intraepithelial lesion, AGC = atypical glandular cells, HSIL = High grade squamous intraepithelial lesion.

## Data Availability

The data generated in the present study may be requested from the corresponding author.

## Supplementary Materials

The following supporting information can be downloaded at: https://www.mdpi.com/article/10.3390/cancers17203328/s1, Figure S1: (A) Flow cytometric gating and p16^INK4a^ and Ki-67 expression of HeLa (cervical cancer cell line, HPV18+) and U2OS (osteosarcoma, HPV−). (B) Quantification of CK8 expression of population 1 from various cancer cell lines; Figure S2: Flow cytometry gating of cervical biofluids and CaSki cells showing EpCAM vs. respective isotype staining; Figure S3: Dot plots and histograms of flow cytometry measurements showing additional tumor samples; Figure S4: Evaluation of Pap samples from normal to various stages cervical neoplasia; Figure S5: Hematoxylin and eosin and immunohistochemical stainings for p16^INK4a^ and Ki-67 of surgically removed tissues of the cervix of patients included in this study; Figure S6: Compensation strategy for flow cytometry done on a four laser CytoFLEX S device.

## References

[1] Mitra T., Elangovan S. (2021). Cervical cancer development, chemoresistance, and therapy: A snapshot of involvement of microRNA. Mol. Cell. Biochem..

[2] Schiffman M., Doorbar J., Wentzensen N., de Sanjose S., Fakhry C., Monk B.J., Stanley M.A., Franceschi S. (2016). Carcinogenic human papillomavirus infection. Nat. Rev. Dis. Primers.

[3] Maru Y., Kawata A., Taguchi A., Ishii Y., Baba S., Mori M., Nagamatsu T., Oda K., Kukimoto I., Osuga Y. (2020). Establishment and Molecular Phenotyping of Organoids from the Squamocolumnar Junction Region of the Uterine Cervix. Cancers.

[4] Hausen H.Z. (2002). Papillomaviruses and cancer: From basic studies to clinical application. Nat. Rev. Cancer.

[5] Han K., Zou J., Zhao Z., Baskurt Z., Zheng Y., Barnes E., Croke J., Ferguson S.E., Fyles A., Gien L. (2024). Clinical Validation of Human Papilloma Virus Circulating Tumor DNA for Early Detection of Residual Disease After Chemoradiation in Cervical Cancer. J. Clin. Oncol..

[6] Kamal M. (2022). Pap Smear Collection and Preparation: Key Points. CytoJournal.

[7] Tanaka L.F., Schoffer O., Schriefer D., Schauberger G., Ikenberg H., Klug S.J. (2024). An audit of 1632 routinely collected cervical cancer screening smears from 398 women in Germany: Results from the TeQaZ Study. Eur. J. Cancer.

[8] Edvardsson H., Wang J., Andrae B., Sparen P., Strander B., Dillner J. (2021). Nationwide Rereview of Normal Cervical Cytologies before High-Grade Cervical Lesions or before Invasive Cervical Cancer. Acta Cytol..

[9] Ling J., Wiederkehr U., Cabiness S., Shroyer K.R., Robinson J.P. (2008). Application of flow cytometry for biomarker-based cervical cancer cells detection. Diagn. Cytopathol..

[10] Grundhoefer D., Patterson B.K. (2001). Determination of liquid-based cervical cytology specimen adequacy using cellular light scatter and flow cytometry. Cytometry.

[11] Kottaridi C., Georgoulakis J., Kassanos D., Pappas A., Spathis A., Margari N., Aninos D., Karakitsos P. (2010). Use of flow cytometry as a quality control device for liquid-based cervical cytology specimens. Cytom. B Clin. Cytom..

[12] Reich O., Braune G., Eppel W., Fiedler T., Graf A., Hefler L., Joura E., Kölbl H., Marth C., Pokieser W. (2018). Joint Guideline of the OEGGG, AGO, AGK and ÖGZ on the Diagnosis and Treatment of Cervical Intraepithelial Neoplasia and Appropriate Procedures When Cytological Specimens Are Unsatisfactory. Geburtshilfe Frauenheilkd..

[13] Litvinov S.V., van Driel W., van Rhijn C.M., Bakker H.A.M., van Krieken H., Fleuren G.J., Warnaar S.O. (1996). Expression of Ep-CAM in Cervical Squamous Epithelia Correlates with an Increased Proliferation and the Disappearance of Markers for Terminal Differentiation. Am. J. Pathol..

[14] Chantima W., Thepthai C., Cheunsuchon P., Dharakul T. (2017). EpCAM expression in squamous cell carcinoma of the uterine cervix detected by monoclonal antibody to the membrane-proximal part of EpCAM. BMC Cancer.

[15] Gires O., Pan M., Schinke H., Canis M., Baeuerle P.A. (2020). Expression and function of epithelial cell adhesion molecule EpCAM: Where are we after 40 years?. Cancer Metastasis Rev..

[16] Wentzensen N., von Knebel Doeberitz M. (2007). Biomarkers in cervical cancer screening. Dis. Markers.

[17] Remnant L., Kochanova N.Y., Reid C., Cisneros-Soberanis F., Earnshaw W.C. (2021). The intrinsically disorderly story of Ki-67. Open Biol..

[18] Senzaki H., Ogura E., Iwamoto S., Nambu H., Uemura Y., Shikata N., Tsubura A. (1997). Keratin expression in normal uterine cervical epithelium and carcinomas of cervical origin. Oncol. Rep..

[19] Ikeda K., Tate G., Suzuki T., Mitsuya T. (2008). Coordinate expression of cytokeratin 8 and cytokeratin 17 immunohistochemical staining in cervical intraepithelial neoplasia and cervical squamous cell carcinoma: An immunohistochemical analysis and review of the literature. Gynecol. Oncol..

[20] Smedts F., Ramaekers F., Troyanovsky S., Pruszczynksi M., Link M., Lane B., Leigh I., Schijf C., Vooijs P. (1992). Keratin Expression in Cervical Cancer. Am. J. Pathol..

[21] Ivanyi D., Groeneveld E., Van Doornewaard G., Mooi W.J., Hageman P.C. (1990). Keratin Subtypes in Carcinomas of the Uterine Cervix: Implications for Histogenesis and Differential Diagnosis. Cancer Res..

[22] Martens J., Baars J., Smedts F., Holterheus M., Kok M.-J., Vooijs P., Ramaekers F. (1999). Can Keratin 8 and 17 Immunohistochemistry Be of Diagnostic Value in Cervical Cytology. Cancer Cytopathol..

[23] Denk C. (2018). Nachweis Zirkulierender Tumorzellen Mittels Durchflusszytometrie bei Patientinnen mit Karzinom der Cervix Uteri. Ph.D. Thesis.

[24] Richter C.E., Cocco E., Bellone S., Bellone M., Casagrande F., Todeschini P., Ruettinger D., Silasi D.-A., Azodi M., Schwartz P.E. (2010). Primary Cervical Carcinoma Cell Lines Overexpress Epithelial Cell Adhesion Molecule (EpCAM) and Are Highly Sensitive to Immunotherapy with MT201, a Fully Human Monoclonal AntiEpCAM Antibody. Int. J. Gynecol. Cancer.

[25] Dovnik A., Repse Fokter A. (2023). The Role of p16/Ki67 Dual Staining in Cervical Cancer Screening. Curr. Issues Mol. Biol..

[26] Howitt B.E., Nucci M.R., Drapkin R., Crum C.P., Hirsch M.S. (2013). Stathmin-1 Expression as a Complement to p16 Helps Identify High-grade Cervical Intraepithelial Neoplasia with Increased Specificity. Am. J. Surg. Pathol..

[27] Imai Y., Fukagawa Y., Sugihara S., Teranishi T. (2022). Reclassification of atypical immature metaplasia of the uterine cervix by combination of nuclear features on hematoxylin and eosin–stained sections without auxiliary immunohistochemistry. Hum. Pathol..

[28] Miller I., Min M., Yang C., Tian C., Gookin S., Carter D., Spencer S.L. (2018). Ki67 is a Graded Rather than a Binary Marker of Proliferation versus Quiescence. Cell Rep..

[29] Bruno S., Darzynkiewicz Z. (1992). Cell cycle dependent expression and stability of the nuclear protein detected by Ki-67 antibody in HL-60 cells. Cell Prolif..

[30] Krtinic D., Zivadinovic R., Jovic Z., Pesic S., Mihailovic D., Ristic L., Cvetanovic A., Todorovska I., Zivkovic N., Rankovic G.N. (2018). Significance of the Ki-67 proliferation index in the assessment of the therapeutic response to cisplatin-based chemotherapy in patients with advanced cervical cancer. Eur. Rev. Med. Pharmacol. Sci..

[31] Carozzi F., Confortini M., Palma P.D., Del Mistro A., Gillio-Tos A., De Marco L., Giorgi-Rossi P., Pontenani G., Rosso S., Sani C. (2008). Use of p16-INK4A overexpression to increase the specificity of human papillomavirus testing: A nested substudy of the NTCC randomised controlled trial. Lancet Oncol..

[32] da Mata S., Ferreira J., Nicolas I., Esteves S., Esteves G., Lerias S., Silva F., Saco A., Cochicho D., Cunha M. (2021). P16 and HPV Genotype Significance in HPV-Associated Cervical Cancer-A Large Cohort of Two Tertiary Referral Centers. Int. J. Mol. Sci..

[33] He Y., Shi J., Zhao H., Wang Y., Zhang C., Han S., He Q., Li X., Li S., Wang W. (2023). p16(INK4A) flow cytometry of exfoliated cervical cells: Its role in quantitative pathology and clinical diagnosis of squamous intraepithelial lesions. Clin. Transl. Med..

[34] Wentzensen N., Schwartz L., Zuna R.E., Smith K., Mathews C., Gold M.A., Allen R.A., Zhang R., Dunn S.T., Walker J.L. (2012). Performance of p16/Ki-67 immunostaining to detect cervical cancer precursors in a colposcopy referral population. Clin. Cancer Res..

[35] Litwin T.R., Irvin S.R., Chornock R.L., Sahasrabuddhe V.V., Stanley M., Wentzensen N. (2021). Infiltrating T-cell markers in cervical carcinogenesis: A systematic review and meta-analysis. Br. J. Cancer..

[36] Li Y., Lu S., Wang S., Peng X., Lang J. (2021). Identification of immune subtypes of cervical squamous cell carcinoma predicting prognosis and immunotherapy responses. J. Transl. Med..

[37] Punt S., Corver W.E., van der Zeeuw S.A., Kielbasa S.M., Osse E.M., Buermans H.P., de Kroon C.D., Jordanova E.S., Gorter A. (2015). Whole-transcriptome analysis of flow-sorted cervical cancer samples reveals that B cell expressed TCL1A is correlated with improved survival. Oncotarget.

[38] Wu Z., Lin Q., Sheng L., Chen W., Liang M., Wu D., Ke Y. (2023). A novel immune-related risk-scoring system associated with the prognosis and response of cervical cancer patients treated with radiation therapy. Front. Mol. Biosci..

[39] Rocha Martins P., Luciano Pereira Morais K., de Lima Galdino N.A., Jacauna A., Paula S.O.C., Magalhaes W.C.S., Zuccherato L.W., Campos L.S., Salles P.G.O., Gollob K.J. (2023). Linking tumor immune infiltrate and systemic immune mediators to treatment response and prognosis in advanced cervical cancer. Sci. Rep..

[40] Wang Y., Li L., Douville C., Cohen J.D., Yen T.-T., Kinde I., Sundfelt K., Kjær S.K., Hruban R.H., Shih I.M. (2018). Evaluation of liquid from the Papanicolaou test and other liquid biopsies for the detection of endometrial and ovarian cancers. Sci. Transl. Med..

