# Supplementary material for: Noninvasive Cell Population Profiling of Normal and Dysplastic Cervical Biofluids by Multicolor Flow Cytometry as a Promising Tool for Companion Diagnostics
Source: Cancers (Basel). 2025 Oct 15;17(20):3328. doi: 10.3390/cancers17203328 (PMC12562902; doi:10.3390/cancers17203328)
Supplement: Supplementary file 1 [file cancers-17-03328-s001.zip › cancers-3880774-supplementary.pdf]

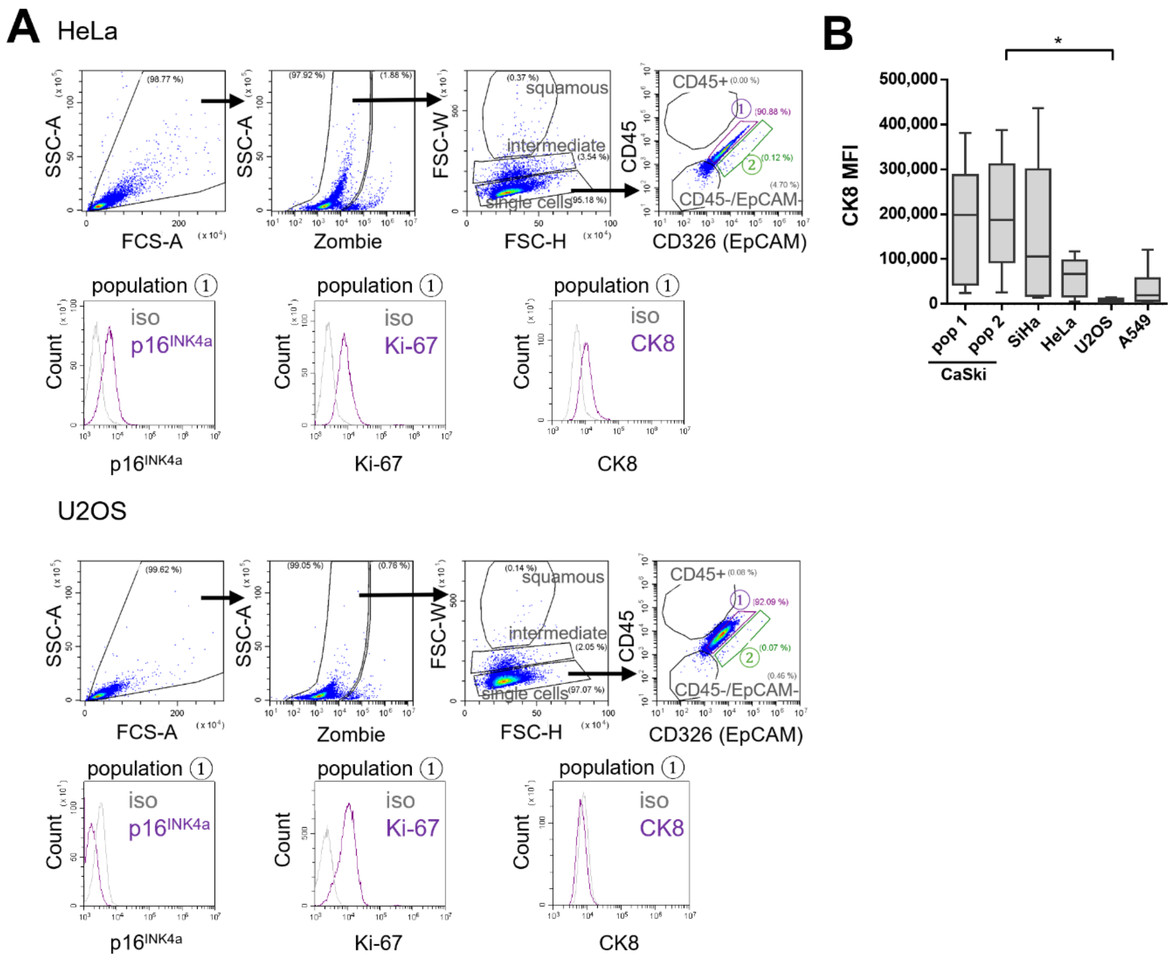

Supplementary Figure S1: (A) Flow cytometric gating and $p16^{INK4a}$ and Ki-67 expression of HeLa (cervical cancer cell line, HPV18+) and U2OS (osteosarcoma, HPV−).

Dot plots and histograms of flow cytometry measurements showing example gating of HeLa and U2OS. From left to right, FSC vs. SSC plot shows gating including “all cells”; SSC vs. Zombie (Fixable Viability Kit) shows the exclusion of dead cells (Zombie positive); FSC-W vs. FSC-H plot shows gate for “single cells” (lowest gate); and CD45 vs. EpCAM plot to gate for immune cells and epithelial (cancer) cells, respectively. Histograms below show $p16^{INK4a}$ and Ki-67 expression of population ① (purple) compared to isotype (iso; grey). No histograms for $p16^{INK4a}$ nor Ki-67 of population ② (green) are shown due to the sparce number of cells in this gate. (B) Quantification of CK8 expression of population (pop) ① from various cancer cell lines (for CaSki also population ②). median (min/max) of n = 6, * $p \leq 0.05$

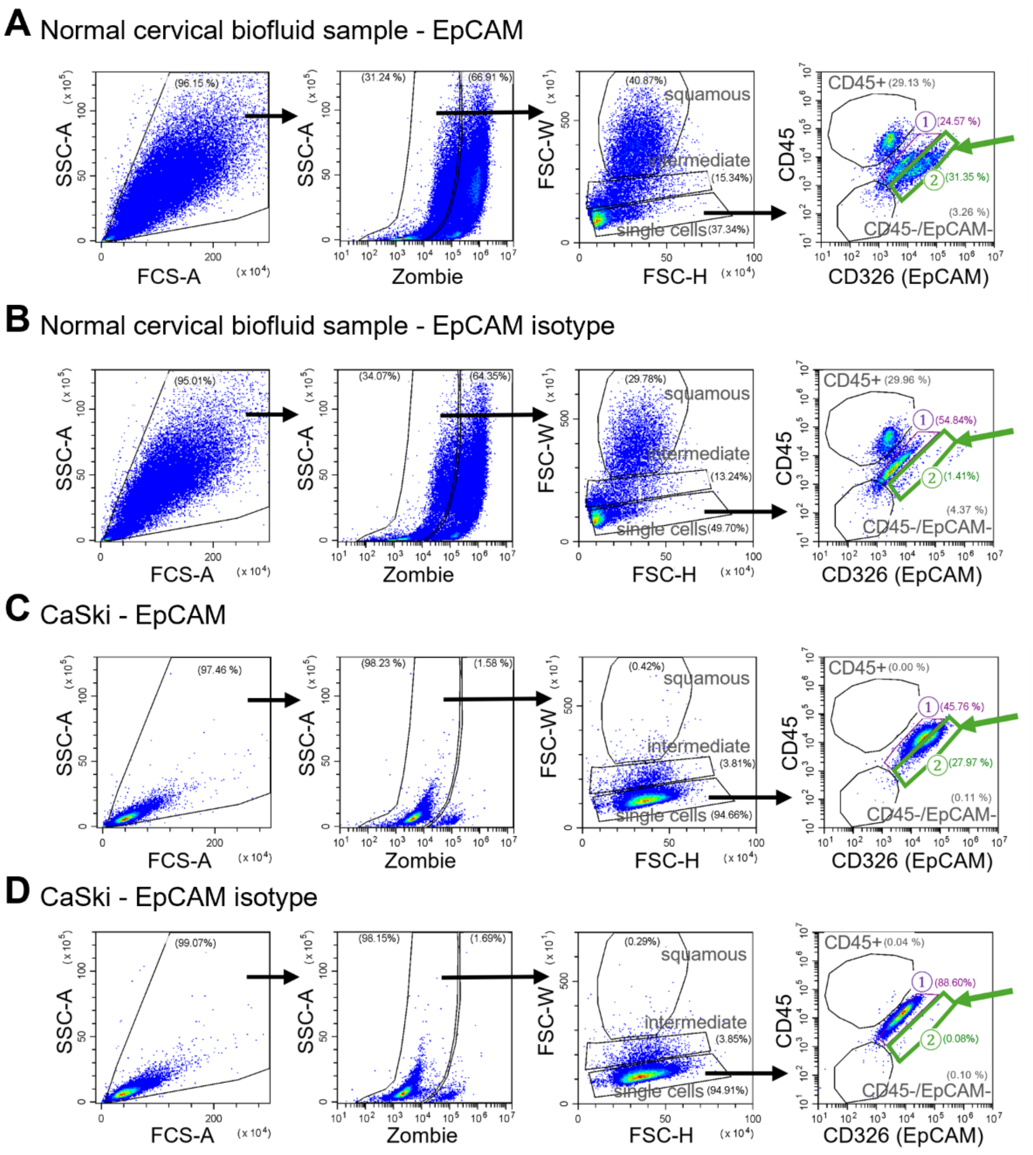

Supplementary Figure S2: Flow cytometry gating of cervical biofluids and CaSki cells showing EpCAM vs. respective isotype staining. From left to right, FSC vs. SSC plot shows gating including “all cells”; SSC vs. Zombie (Fixable Viability Kit) shows the exclusion of dead cells (Zombie positive); FSC-W vs. FSC-H plot shows gate for “single cells” (lowest gate); and CD45 vs. EpCAM plot showing in green the EpCAM positive population (isotype staining was used as negative control to adjust gating). (A, B) A cervical biofluid sample diagnosed as Pap II/NILM and without CIN or inflammation is illustrated. (C, D) CaSki cell line samples are depicted. (B, D) show isotypes, whereas (A, C) show samples stained with labelled antibody.

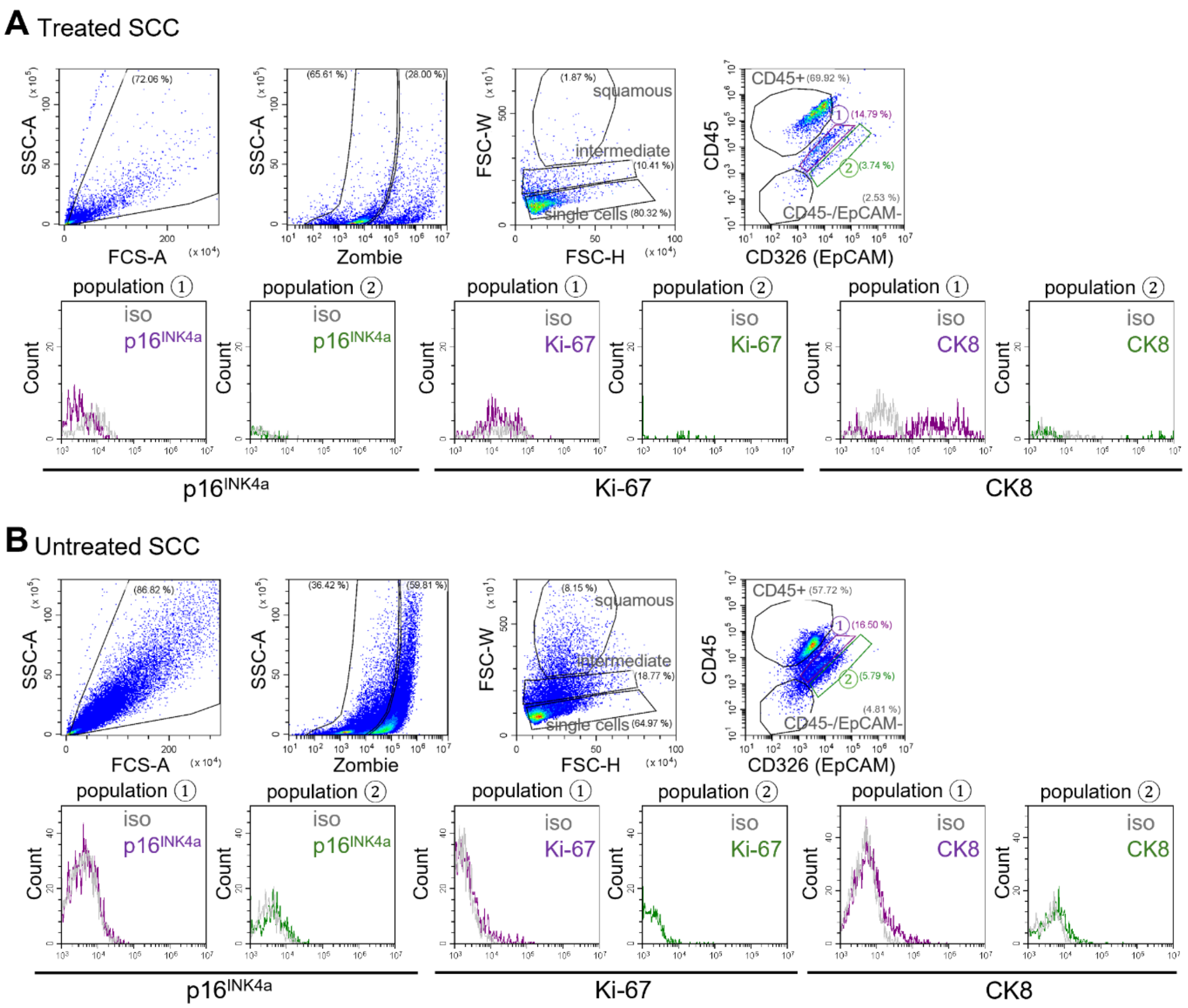

Supplementary Figure S3: Dot plots and histograms of flow cytometry measurements showing additional tumor samples.

From left to right, FSC vs. SSC plot shows gating including “all cells”; SSC vs. Zombie (Fixable Viability Kit) shows the exclusion of dead cells (Zombie positive); FSC-W vs. FSC-H plot shows gate for “single cells” (lowest gate); and CD45 vs. EpCAM plot to gate for immune cells and epithelial (cancer) cells, respectively. Histograms below show $p16^{INK4a}$, Ki-67 and CK8 expression of population ① (left, purple) and population ② (right, green) compared to isotype (iso; grey). (A) SCC biofluid sample of a chemo-radiotherapy treated women. (B) SCC from an untreated woman.

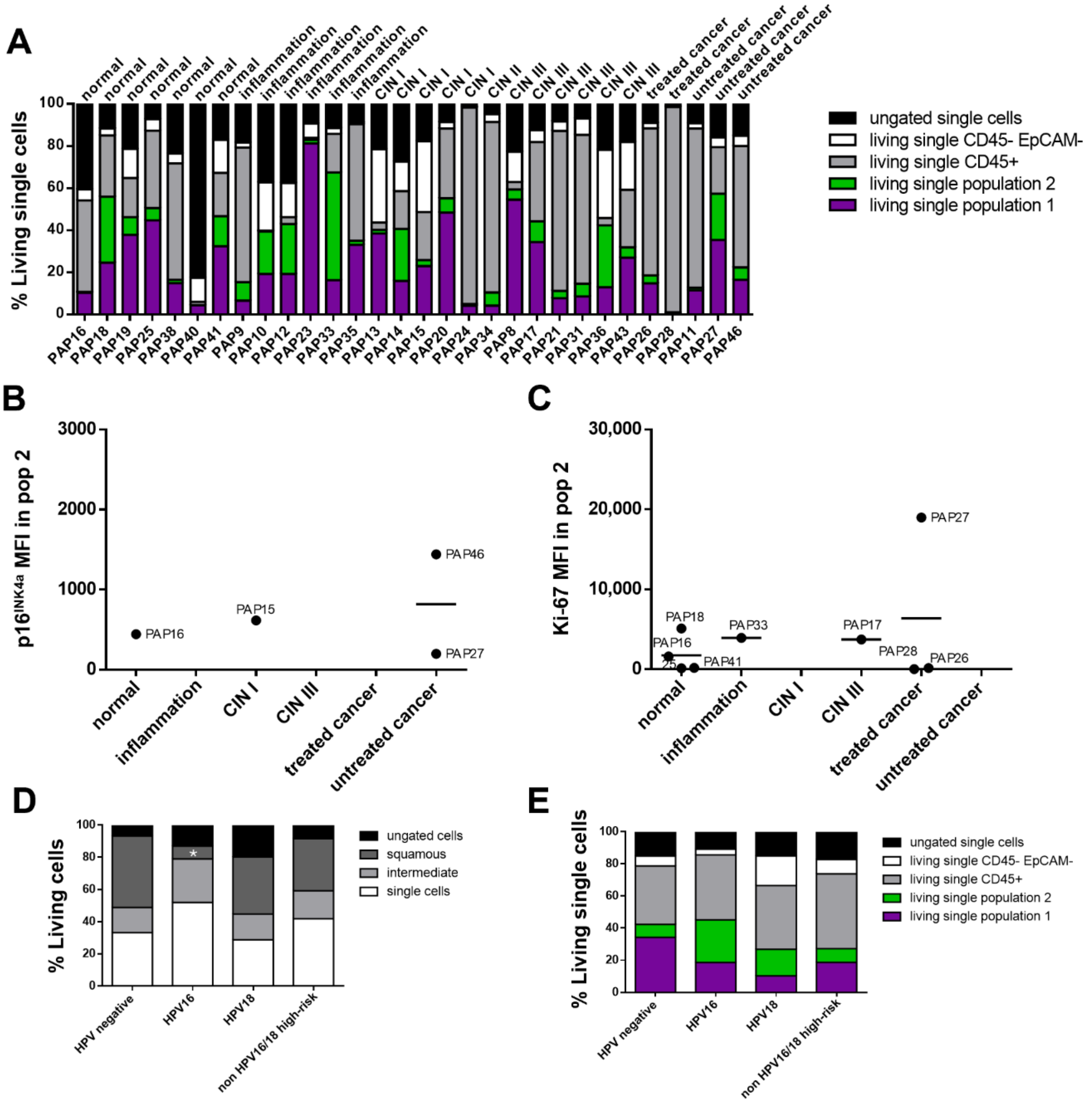

Supplementary Figure S4: Evaluation of Pap samples from normal to various stages cervical neoplasia.

(A) Individual Pap smear samples sorted according to disease and stage. Of each sample, “CD45+”, “CD45−/EpCAM−“, “population 1” and “population 2” are separated according to CD45 vs. EpCAM staining. (B, C) Samples with expression higher than isotype of p16^{INK4a} (B) or Ki-67 (C) in population 2 are shown grouped by disease stage. Pap samples are numbered chronologically based on time of receival. (D, E) Samples stratified based on their HPV subtype identified via qPCR. Significance vs. HPV negative $p \leq 0.05$ (*)

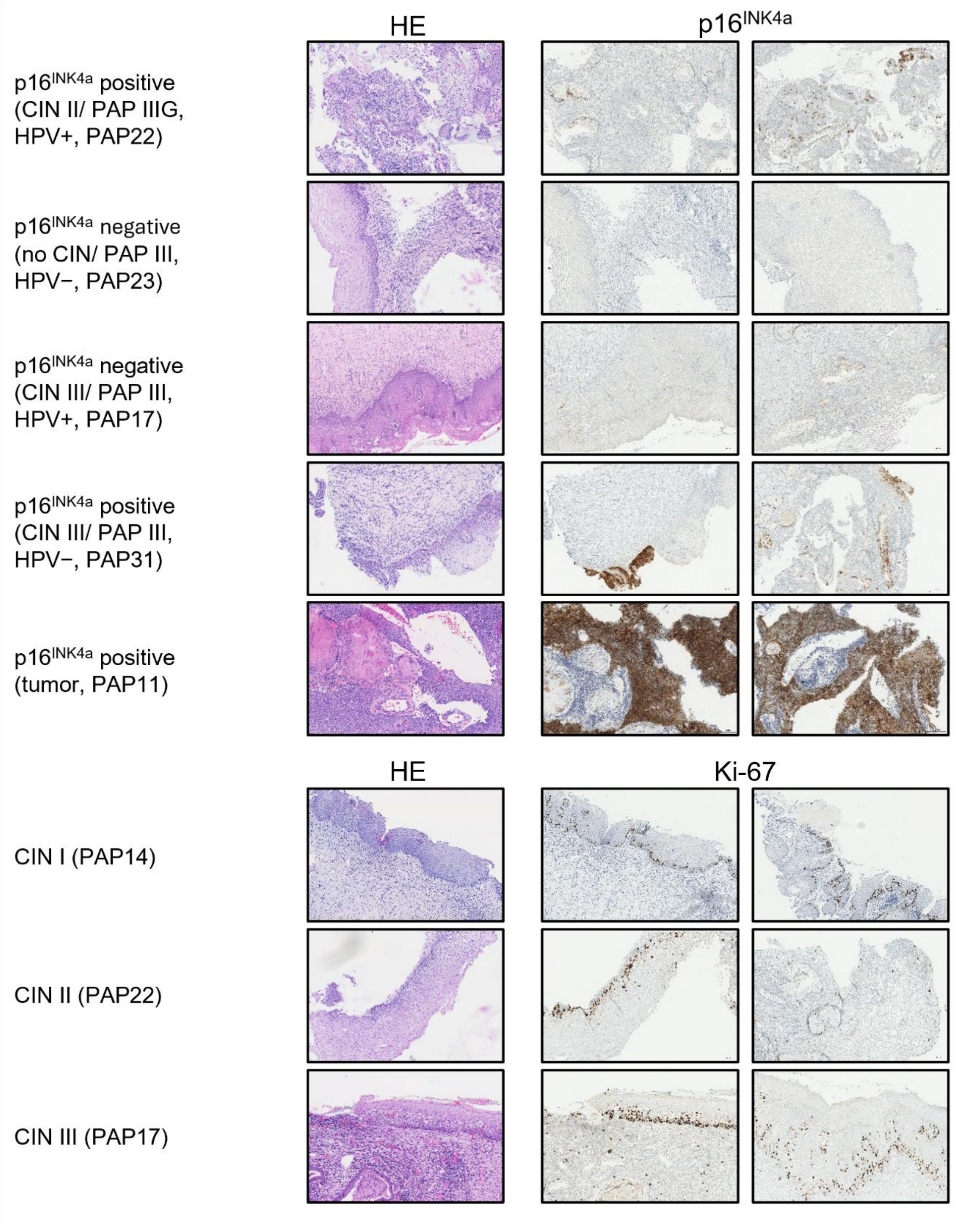

Supplementary Figure S5: Hematoxylin and eosin and immunohistochemical (IHC) stainings for p16^{INK4a} and Ki-67 of surgically removed tissues of the cervix of patients included in this study. Two regions of the IHC stained tissues are shown. HPV and p16^{INK4a} status as well as disease stage and sample ID are indicated.

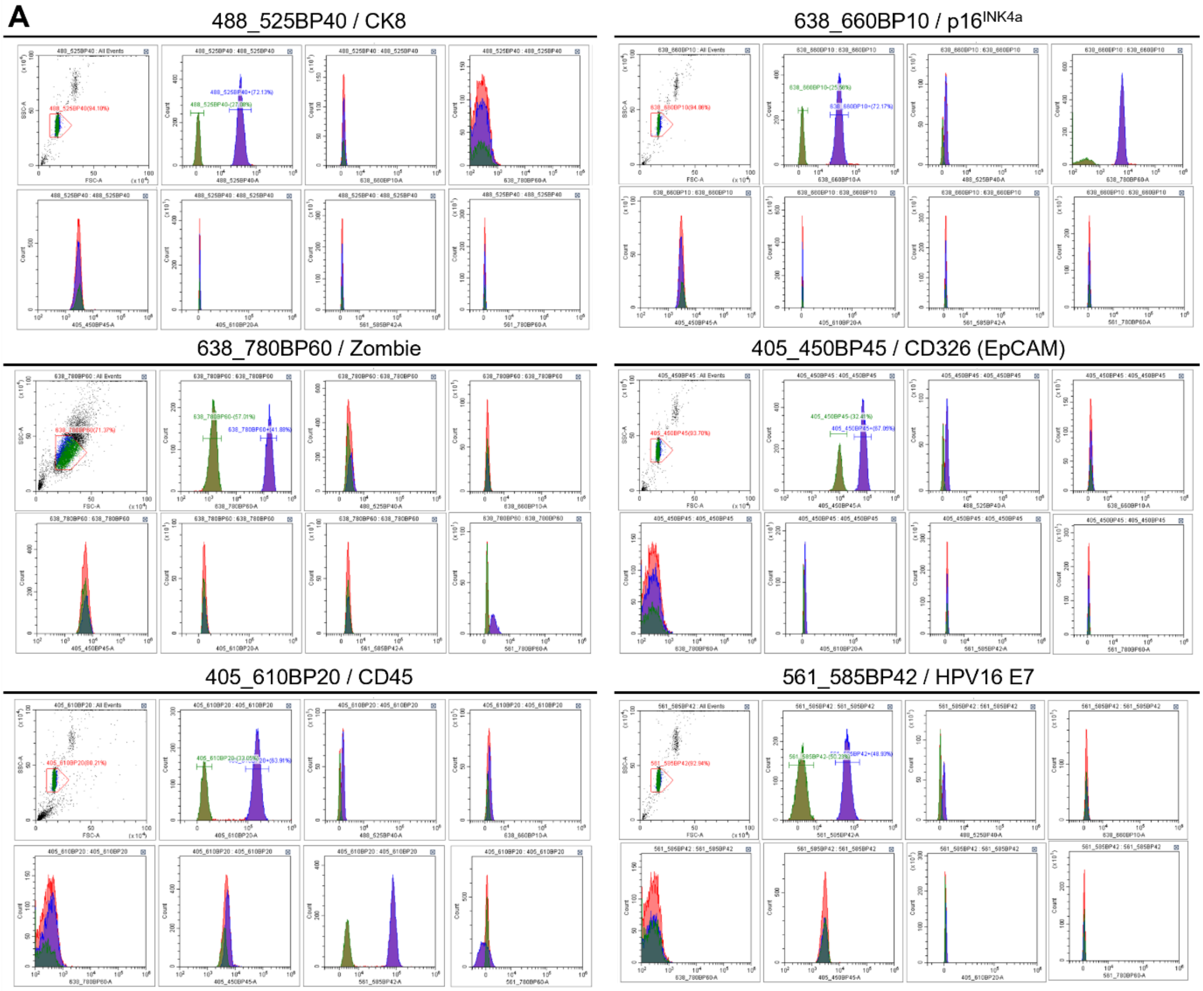

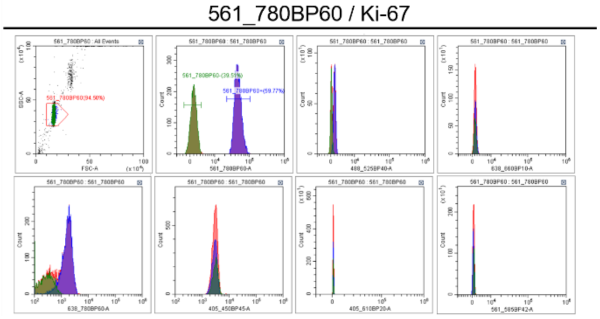

B

Channel	488_525BP50 %	638_660BP10 %	638_780BP60 %	405_450BP45 %	405_610BP20 %	561_585BP42 %	561_780BP60 %
488_525BP50 %		-	-	-	-	-	-
638_660BP10 %	-		0.56	-	-	-	-
638_780BP60 %	-	-		-	-	-	5.35
405_450BP45 %	-	-	-		3.13	-	-
405_610BP20 %	-	-	-	-		-	-
561_585BP42 %	-	-	-	-	-		2.98
561_780BP60 %	-	-	8.26	-	4.73	0.6	

Supplementary Figure S6: Compensation strategy for flow cytometry done on a four laser CytoFLEX S device.

(A) Compensation beads (AbC™ Total Antibody Compensation Beads) were stained with the respective antibody for each fluorescence channel. Beads without antibody conjugate capture capacity were used as negative control. For 638_780BP60 compensation Zombie positive and negative stained cell lines were used (A549 are shown as a representative cell line). A group of flow cytometry plots is shown for the different antibody labeled compensation beads and the according fluorescence channel. First graph of each channel shows the SSC vs. FSC plot where gating was performed to resemble the beads. The other seven graphs depict histograms for different fluorescence channels to indicate the compensation. The HPV16 E7 antibody detected in channel 561_585BP42 was not further analyzed for this study. Compensation matrices were generated automatically using CytExpert and verified manually to ensure accuracy.
(B) Final compensation matrix.
